# Activation of Inflammation/NF-κB Signaling in Infants Born to Arsenic-Exposed Mothers

**DOI:** 10.1371/journal.pgen.0030207

**Published:** 2007-11-23

**Authors:** Rebecca C Fry, Panida Navasumrit, Chandni Valiathan, J. Peter Svensson, Bradley J Hogan, Manlin Luo, Sanchita Bhattacharya, Krittinee Kandjanapa, Sumitra Soontararuks, Sumontha Nookabkaew, Chulabhorn Mahidol, Mathuros Ruchirawat, Leona D Samson

**Affiliations:** 1 Department of Biological Engineering, Massachusetts Institute of Technology, Cambridge, Massachusetts, United States of America; 2 Center for Environmental Health Sciences, Massachusetts Institute of Technology, Cambridge, Massachusetts, United States of America; 3 Chulabhorn Research Institute, Bangkok, Thailand; University of Pennsylvania, United States of America

## Abstract

The long-term health outcome of prenatal exposure to arsenic has been associated with increased mortality in human populations. In this study, the extent to which maternal arsenic exposure impacts gene expression in the newborn was addressed. We monitored gene expression profiles in a population of newborns whose mothers experienced varying levels of arsenic exposure during pregnancy. Through the application of machine learning–based two-class prediction algorithms, we identified expression signatures from babies born to arsenic-unexposed and -exposed mothers that were highly predictive of prenatal arsenic exposure in a subsequent test population. Furthermore, 11 transcripts were identified that captured the maximal predictive capacity to classify prenatal arsenic exposure. Network analysis of the arsenic-modulated transcripts identified the activation of extensive molecular networks that are indicative of stress, inflammation, metal exposure, and apoptosis in the newborn. Exposure to arsenic is an important health hazard both in the United States and around the world, and is associated with increased risk for several types of cancer and other chronic diseases. These studies clearly demonstrate the robust impact of a mother's arsenic consumption on fetal gene expression as evidenced by transcript levels in newborn cord blood.

## Author Summary

Arsenic is an environmental pollutant and known human carcinogen. Chronic exposure to arsenic-contaminated water is an important public health hazard around the world, including the United States, with millions exposed to drinking water with levels that far exceed World Health Organization (WHO) guidelines. Given the implications of prenatal exposure on human health and the known public health hazard of chronic arsenic exposure, this study was aimed at establishing the extent to which maternal arsenic exposure in a human population affects newborn gene expression. The authors show that prenatal arsenic exposure in a human population results in alarming gene expression changes in newborn babies. The gene expression changes monitored in babies born to mothers exposed to arsenic during pregnancy are highly predictive of prenatal arsenic exposure in a subsequent test population. The study establishes a subset of just 11 transcripts that captured maximal predictive capability that could prove promising as genetic biomarkers of prenatal arsenic exposure. Pathway analysis of the genome-wide response in the babies exposed to arsenic in utero indicates robust activation of an integrated network of pathways involving NF-κB, inflammation, cell proliferation, stress, and apoptosis. This study contributes to our understanding of biological responses to arsenic exposure.

## Introduction

Arsenic is a ubiquitous environmental pollutant and a known human carcinogen [1]. Chronic arsenic exposure is an important public health hazard around the world, with millions of people exposed to drinking water with levels far exceeding the guideline of 10 μg/l established by the WHO. Exposure to arsenic-contaminated drinking water is alarmingly high in many countries, most notably Bangladesh, where >25 million people are chronically exposed to extreme arsenic levels. Arsenic contamination is also a significant health concern in the United States, with numerous public water supplies measuring above the WHO limit [2].

Epidemiological studies indicate that chronic arsenic exposure in drinking water is associated with increased risk of skin, bladder, lung, liver, and kidney cancer [1]; in 1987, arsenic was classified as a Group 1 carcinogen by the International Agency for Research on Cancer. Although the mechanism of arsenic-induced carcinogenesis is not clearly established, it has been attributed to genotoxicity associated with reactive oxygen species [3]. Arsenic is also implicated in other human diseases such as vascular disorders, peripheral neuropathy, bronchiecstasis, and diabetes [1].

The long-term health consequences of prenatal arsenic exposure in human populations are pronounced, with increased mortality rates caused by prenatal and early childhood exposures [4]. The detrimental health impact of prenatal arsenic exposure has also been shown in rodent models where in utero arsenic exposure resulted in a striking carcinogenic response (5-fold increase in hepatocellular carcinomas) among offspring; in utero arsenic exposure also changed the expression of genes involved in cell proliferation, stress, and cell–cell communication that are evident even when the offspring reach adulthood. These results have profound implications suggesting that in utero arsenic exposure may result in epigenetic changes that persist through the life of the organism, ultimately impacting health status. A landmark study in mouse models shows that, indeed, in utero exposures via the maternal diet can cause permanent gene expression changes in the offspring that affect susceptibility to disease in the adult [7].

Given the implications of prenatal exposure on human health and the known public health hazard of chronic arsenic exposure, we set out to establish the extent to which maternal arsenic exposure in a human population impacts newborn gene expression. Additionally, these studies were aimed at understanding exactly how arsenic affects biological systems and identifying genes that could be used as predictors, and therefore potential biomarkers, of prenatal arsenic exposure.

## Results

Our study was based in the Ron Pibul and Bangkok districts of Thailand (Figure S1). The first case of arsenicosis (arsenic poisoning) in Thailand was reported in 1987 from the Ron Pibul district [8]. Rather than natural leaching of arsenic from geologic sources, Ron Pibul arsenic contamination is attributed to tin mining that took place from the 1960s to the 1980s. Arsenic concentrations in groundwater and shallow wells have been classified at a mean level of 503.5 μg/l, about 50 times higher than WHO guidelines [9].

**Figure 1:**
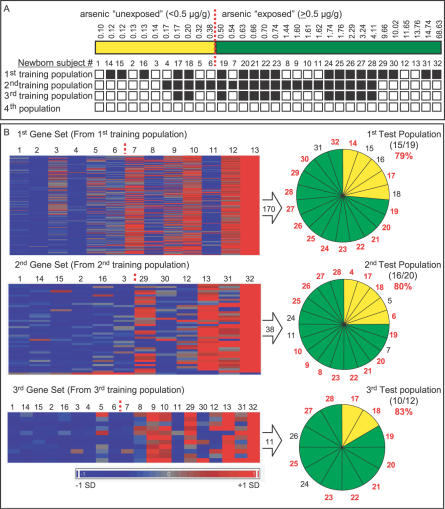
Gene Expression Signatures Predict Arsenic Exposure in Test Populations (A) A population of newborns (subjects 1–32) born to mothers with varying levels of arsenic exposure was used to establish arsenic-associated gene expression signatures. Arsenic exposure levels were determined by maternal toenail arsenic concentration (μg/g). Babies born to unexposed (yellow) or arsenic-exposed mothers (green) were classified based on WHO guidelines with the cut point demarcated by the red dotted line. Subjects used in the populations to establish arsenic-associated gene sets are indicated with a white box. For two-class prediction, those subjects not included in the training population comprise the test population and are indicated with a black box. (B) Three arsenic expression signatures (gene sets) were derived from populations spanning the range of arsenic exposure (first gene set), at the extremes of exposure (second gene set), or a combined population of the previous two (third gene set). To be included in the gene set, the transcript had to not only be differentially expressed (on average) between the exposed and unexposed groups, but also display a significant trend across increasing arsenic exposure levels. Expression values are mean centered with high relative expression indicated in red and low relative expression indicated in blue. The three derived gene sets (170 genes, 38 genes, or 11 genes) were used to predict prenatal arsenic exposure in test populations where correct classification is indicated by a red number.

Using a population of arsenic-exposed and -unexposed mothers (as defined by WHO standards of chronic exposure to ∼10 μg/l arsenic), we set out to identify gene expression changes in the cord blood of newborns significantly associated with the extent of prenatal arsenic exposure. Cord blood is derived almost exclusively from the fetus; therefore, gene expression changes assessed in cord blood are representative of the newborn [10]. For this study, exposure classification was based on arsenic concentration in the mother's toenails, as this is representative of long-term arsenic accumulation [11,12]. Toenail samples were taken from a population of 32 volunteer subjects to quantify arsenic exposure in the mothers. A level of 0.5 μg/g toenail arsenic corresponds to chronic consumption of water with ∼10 μg/l (see Materials and Methods), which is the official WHO maximum recommended concentration of arsenic in drinking water [11,12]. For the purposes of this study, women with toenail arsenic levels of <0.5 μg/g were considered unexposed, and women with toenail levels of ≥0.5 μg/g were considered exposed. The levels of toenail arsenic across the 32 pregnant women ranged from 0.1 to 68.63 μg/g (Figure 1A). Given the paucity of available unexposed newborn cord blood from Ron Pibul, the experimental design required additional utilization of unexposed newborn cord blood samples from Bangkok.

### Gene Expression Signatures Highly Predictive of Arsenic Exposure

We set out to determine whether gene expression changes in a set of infants born to arsenic-exposed women versus unexposed women (as judged by WHO guidelines) could be used to predict arsenic exposure in a test population. For these analyses, two-class prediction was employed, where a training population was used to derive gene sets that were then tested as predictors of exposure in a separate population. The analyses were carried out in two phases: (i) where the training population was selected at random and the analyst “blinded” to arsenic exposure level in the test population and (ii) where all arsenic exposure levels of the population were revealed and used to define new training populations.

The first training population comprised 13 newborn subjects selected at random from the 32 newborns (Figure 1A). Specifically, RNA was extracted from cord blood of newborns 1–13, and hybridized to whole human genome arrays (Materials and Methods). To identify genes whose expression was associated with prenatal arsenic exposure, we used an approach that combined differential expression testing between the populations, plus a positive or inverse correlation of expression with increasing arsenic exposure (Materials and Methods). From the 13 newborn subjects, we identified the first expression signature (first gene set, Figure 1B) composed of 170 genes (Table S1) that differentiated the unexposed newborns (subjects 1–6) from the arsenic-exposed newborns (subjects 7–13). This prenatal arsenic exposure expression signature of 170 genes was then used to predict prenatal exposure in the remaining population of 19 newborns (subjects 14–32). The percent accuracy of class prediction was determined post-analysis by revealing the arsenic exposure of the test population to the analyst. Expression of these 170 genes accurately predicted prenatal arsenic exposure in 15 of 19 (79%) of the newborns (Figure 1B).

When the arsenic levels of the entire population were revealed, it became apparent that the first training population was composed of newborns with a wide range of exposure levels distributed over almost the entire range (Figure 1B). We hypothesized that a training population based on extreme exposures might yield higher predictive capacity. To assess this, arsenic-associated genes were identified using newborns at the extremes of arsenic exposure (i.e., the lowest versus the highest exposures) as the second training population (Figure 1A, second training population). Six newborns comprised the low-exposure population (subjects 1, 14, 15, 2, 16, and 3), and six newborns comprised the high-exposure population (subjects 29, 30, 12, 13, 31, and 32) (Figure 1A). As with the first gene set, differential expression testing and correlation analysis identified an expression signature, this time composed of 38 genes (Table S2) that differentiated infants born to mothers with very low and very high arsenic exposure levels (Figure 1A). These 38 genes were used to predict arsenic exposure in the remaining test population of 20 newborns. Even though the gene set was smaller (38 versus 170), prediction was just as high as that of the first gene set, with prenatal arsenic exposure accurately predicted in 16 of 20 (80%) of the newborns (Figure 1B, second test population).

We next determined whether a training population derived from a combination of all of the training samples used to generate the first and second gene set would yield an expression signature with higher predictive capacity. This third training population was composed of nine unexposed newborns and 11 exposed newborns (Figure 1A). Differential expression testing and correlation analysis identified an expression signature of 11 genes (Figure 1B) that could predict prenatal arsenic exposure in 10 of 12 (83% accuracy) of the remaining newborn test population (Figure 1B). It is noteworthy that with only 11 genes, the power of prediction is as high as the first and second gene sets.

Many of the genes in the third gene set were represented in the gene sets derived from the first and second training populations. Specifically, five of the 11 were identified in the first gene set and all 11 were present in the second gene set (Table 1). Given the high predictive capacity of these 11 genes, we hypothesize that these are key genes involved in the prenatal response of babies to arsenic and represent potential biomarkers of arsenic exposure. The potential arsenic biomarker set is composed of transcripts for the *CXL1*, *DUSP1*, *EGR-1*, *IER2*, *JUNB*, *MIRN21*, *OSM*, *PTGS2*, *RNF149*, *SFRS5*, and *SOC3* genes (Table 1). The dose response of expression level of each of the identified biomarkers is evident when plotted versus arsenic exposure across the population (Figure S2). Furthermore, to substantiate the association of the expression of the biomarkers with arsenic exposure, a multivariate model was employed (Materials and Methods). The model was employed to determine significance of association of expression with two factors: (i) arsenic exposure and (ii) geographic source of samples (Materials and Methods). Geographic source was determined to be a nonsignificant factor for the expression level of the biomarkers (*p* = 0.11), whereas arsenic exposure was determined to be a highly significant factor (*p* = 1.3 × 10^−9^). Furthermore, for the set of biomarkers, the two factors of arsenic exposure and geographic source were not associated (*p* = 0.77).

**Table 1:**
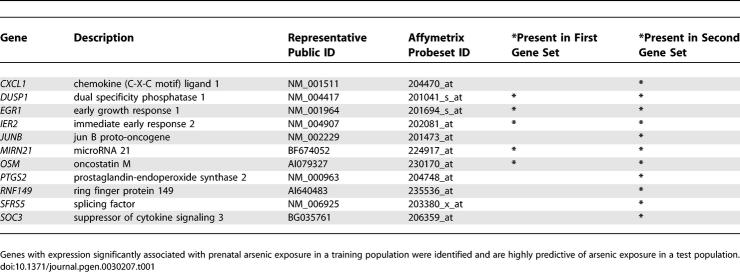
Potential Gene Biomarkers of Prenatal Arsenic Exposure—Third Gene Set

Notably, associated molecular functions for the 11 gene products include stress response and cell cycle regulation. The zinc finger DNA binding transcription factor *EGR-1* (early growth response 1) is related to cell proliferation and is induced by mitogens such as EGF [13]. *EGR-1* regulates both proinflammatory cytokine activation and p53 transcription [14,15]. Not surprisingly, as *EGR-1* is known to activate cytokines, such signaling molecules are present in the arsenic biomarker gene set; namely, *OSM* (oncostatin M), a member of the interleukin-6 (IL-6) family of cytokines known to control cell cycle progression [16], *CXL1* (chemokine ligand 1), and *SOC* (suppressor of cytokine signaling 3). Additionally, *DUSP1* (dual specificity phosphatase 1) is involved in cell cycle regulation and is known to modulate cytokine expression [17,18]. An inflammation-activated acute phase response is indicated by the presence of the *JUNB* transcription factor, and *IER2* (immediate early response 2) transcripts in the biomarker set.

### Genome-Wide Changes Associated with Prenatal Arsenic Exposure Are Robust

**Figure 2:**
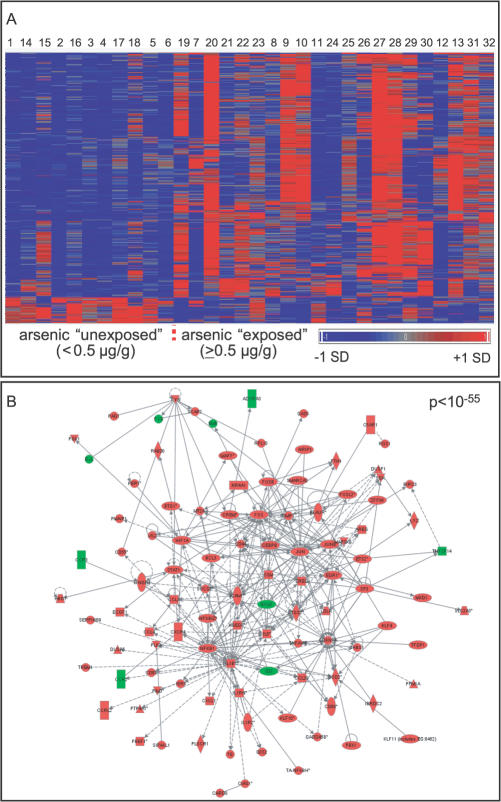
Prenatal Arsenic Exposure Results in Robust Genome-Wide Changes (A) Heat map of the 447 differentially expressed genes identified between two newborn populations, those born to unexposed or arsenic exposed mothers. The cut point of exposure is indicated with a red dotted line. Unlike Figure 1, the differentially expressed transcripts did not have to display a significant trend with increasing arsenic exposure. Expression values are mean centered with high relative expression indicated in red and low relative expression indicated in blue. (B) The 285 arsenic-modulated gene products existing in the Ingenuity database were analyzed for significant enrichment of molecular interactions. A significant (*p* < 10^−55^) interactome containing 105 arsenic-modulated gene products was identified. Proteins in red represent arsenic-induced transcripts, proteins in green represent arsenic-repressed transcripts.

For a more global assessment of the impact of prenatal arsenic exposure on fetal gene expression, all biological pathways modulated in response to arsenic exposure were identified by studying the ontology of all the genes differentially expressed between the exposed and unexposed newborns across the entire population. For these analyses, the entire newborn population was used (the fourth population, Figure 1A) to define the fourth gene set that was differentially expressed between the two populations: the 21 newborns whose mothers were exposed to arsenic and the 11 newborns whose mothers were unexposed. It should be noted that for this analysis of global changes between the populations, the requirement for correlation with increasing arsenic exposure was not imposed (Materials and Methods). This analysis identified 447 genes differentially expressed between the two populations of newborns, of which 404 (90%) were upregulated (Figure 2A; Table S3). Gene ontology enrichment analysis was performed to classify the genes modulated by prenatal arsenic exposure (Materials and Methods). This analysis identified ten gene ontology categories that were significantly enriched in the list of 447 genes (Table 2). Among the gene ontology categories that are significantly enriched are immune and inflammatory response (*p* < 0.001) (Table 2).

**Table 2:**
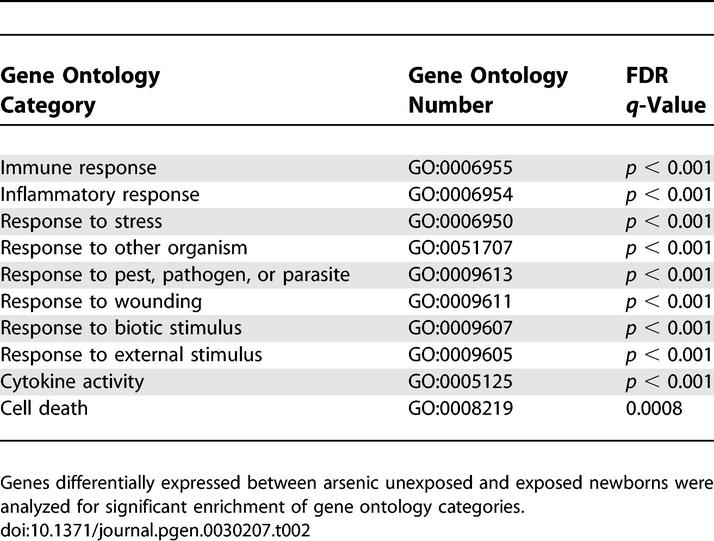
Gene Ontology Enrichment

As an alternative approach to determine if groups of genes with common function are differentially expressed between the two newborn populations (arsenic exposed or unexposed), we have employed the knowledge-based Gene Set Enrichment Analysis (GSEA) (Materials and Methods). GSEA identified significant enrichment (false discovery rate [FDR] *q*-value < 0.01) of ten expression signatures with common biological function that are differentially expressed between the unexposed and exposed newborns. The groups of genes include three that represent stress-response signatures and three that represent tumor/cancer signatures (Table 3). The GSEA results also highlight that genes associated with estrogen receptor signaling are differentially expressed between the unexposed and exposed newborn populations (Table 3).

**Table 3:**
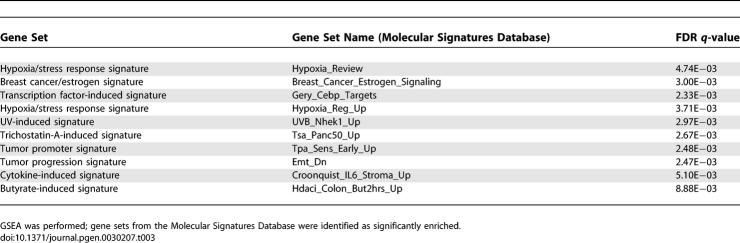
GSEA

### Arsenic-Modulated Networks Represent Numerous Biological Processes

We next determined whether known molecular interactions exist among the proteins encoded by the arsenic modulated transcripts. Of the 447 arsenic modulated transcripts, 285 gene products were identified in the Ingenuity knowledge base and overlayed with known human molecular interactions (Materials and Methods). Among these proteins, we identified the presence of a large arsenic-modulated interacting network of proteins (Figure 2B). Specifically, we identified a large interacting network comprised of 105 human proteins encoded by arsenic-modulated transcripts (indicated as red and green nodes) (Figure 2B; Table S4). The probability of finding 105 arsenic-modulated transcripts that encode for a protein network of this size by chance is *p* < 10^−55^. Of the 105 proteins, 96 (91%) had transcripts that were upregulated in response to arsenic exposure.

**Figure 3:**
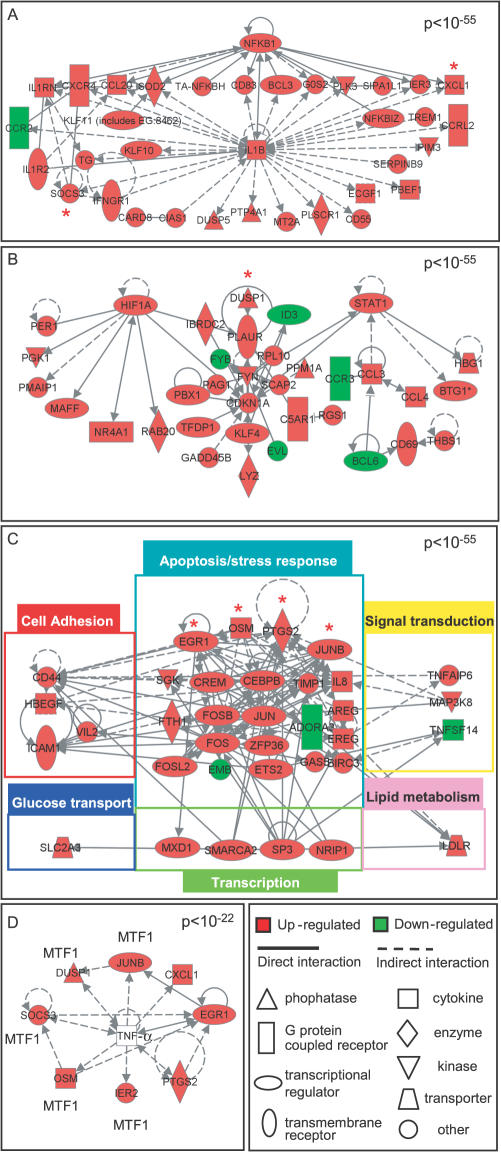
Sub-networks of Prenatal Arsenic-Modulated Gene Products (A) A sub-network that integrates NF-κB and IL1-β with SOC3 and CXCL1 was identified. Note that *SOC3* and *CXCL1* are among the 11 potential gene biomarkers for arsenic exposure shown in Table 1. (B) A sub-network that integrates STAT1 and HIF1-α with DUSP1. (C) An EGR-1, OSM, JUNB focused sub-network highlights numerous biological processes modulated in response to arsenic. Proteins encoded by 11 potential gene biomarkers for arsenic exposure are indicated with a red asterisk. (D) TNF-α–associated network composed of eight core members of the potential gene biomarkers for arsenic exposure. Biomarker genes with binding sites for MTF transcription factor are indicated. Proteins in red represent arsenic-induced transcripts, proteins in green represent arsenic-repressed transcripts.

Further analysis identified three highly significant (*p* < 10^−55^) sub-networks embedded within the large interacting network (Figure 3A–3C). The first sub-network centers around the nuclear transcription factor NF-κB and the pro-inflammatory interleukin 1 family member IL1-β (Figure 3A). This network integrates two members of the potential biomarkers; namely, SOC3 and CXCL1 (Figure 3A). Note that transcripts for all proteins directly associated with NF-κB in this sub-network are upregulated in infants born to arsenic-exposed mothers (Figure 3A).

The second sub-network integrates biomarker member DUSP1 with two stress-activated transcription factors; namely, signal transducer and activator of transcription (STAT1) and hypoxia inducible factor-1 α (HIF-1α) (Figure 3B). Transcripts for both *STAT1* and *HIF-1α* were upregulated in infants with arsenic-exposed mothers (Figure 3B). STAT1 is involved in cytokine signal transduction and is known to be activated by arsenic [19]. HIF-1α activation and resultant tumorigenesis has been linked to chronic arsenic exposure [20].

The third sub-network integrates four of the 11 potential arsenic biomarkers; namely, EGR-1, OSM, PTGS2, and JUNB (Figure 3C). These arsenic biomarker gene products are highly integrated with proteins known to be involved in cell cycle regulation, including JUN and FOS, as well as stress-response proteins such as interleukin-8 (IL-8) (Figure 3C). An overlay of molecular processes represented in this sub-network highlights the finding that prenatal arsenic exposure modulates numerous biological processes including stress response, signal transduction, cell adhesion, and transcription (Figure 3C).

Using network analyses, we also established that there are known molecular interactions among the 11 potential arsenic biomarker genes. Eight of the 11 biomarker gene products (exclusive of SFRS5, MIRN21, and RNF149) are highly integrated with tumor necrosis factor-α (TNF-α), another proinflammatory cytokine (Figure 3D). TNF-α is involved in the control of both cell proliferation and apoptosis [21]. Here, we identify TNF-α activation in newborn cord blood upon exposure to prenatal arsenic.

### Evidence for Arsenic-Activated Transcriptional Control of Prenatal Responses

In an effort to uncover potential regulatory mechanisms underlying the transcription of the arsenic-modulated gene sets, we performed transcription factor binding site analysis within the promoters of the arsenic-modulated genes (Materials and Methods). Promoter region comparisons for the arsenic-modulated genes identified significant enrichment (*p* < 0.05) for two transcription factor binding sites across all four gene sets. Specifically, binding sites for NF-κB and serum response factor (SRF) are enriched in all four arsenic-modulated gene sets (Table 4). Moreover, metal response element binding sites (MREs) for the metal-responsive transcription factor-1 (MTF1) are enriched in three of the four gene sets (sets 1, 3, and 4) (Table 4). The MTF1 binding site enrichment was highest for the third gene set with five of the 11 genes containing the MRE element (Figure 3D). Notably, the enrichment for MTF1 in the second gene set only narrowly misses the enrichment *p* < 0.05 cutoff, at *p* = 0.054 (Table 4). MTF1 was shown to be activated upon arsenic exposure in animal models [23,24]. It is noteworthy that gene targets for a known arsenic-inducible transcription factor are found among the transcripts modulated in the cord blood of infants born to arsenic exposed mothers.

**Table 4:**
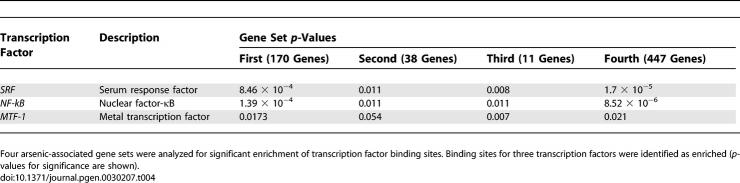
Transcription Factor Binding Site Enrichment

### NF-κB and Inflammation Signaling Identified in Arsenic-Exposed Newborns from Ron Pibul and Common Arsenic-Induced Stress Signaling across Species

As the unexposed samples utilized in this study were obtained from two different locations and could confound expression testing, we have used an alternative approach to substantiate the identified arsenic-induced pathways. Differential expression testing was performed between the cord blood of exposed and unexposed newborns from Ron Pibul (Materials and Methods). These analyses identified 321 genes that were differentially expressed between the arsenic-unexposed and -exposed newborns (Table S5). Notably, a direct comparison of gene expression changes identified considerable overlap between the transcripts differentially expressed between the newborns from Ron Pibul and transcripts differentially expressed across the whole population (fourth gene set) (Table S5).

To identify the biological pathways modulated by prenatal arsenic exposure, the proteins encoded by the 321 transcripts were analyzed for significant enrichment of molecular networks (Materials and Methods). Three highly significant protein sub-networks (*p* < 10^−30^) were identified (Figure S3). As with the network findings from the entire population of newborns, the networks identified here integrate proteins known to be involved in cell cycle regulation including JUN, as well as stress-response proteins such as interleukin-8 (IL-8), the pro-inflammatory interleukin 1 family member IL1-β, and hypoxia inducible factor-1 α (HIF-1α) (Figure S3). Furthermore, the NF-κB protein is integrated into the sub-networks and found to be activated in the cord blood of newborns exposed to arsenic within the Ron Pibul population (Figure S3).

Finally, our analyses included comparisons of the gene expression changes identified in this study with arsenic-induced gene expression changes reported in the literature in mouse models as well as a separate arsenic-exposed human population. Our results were compared with (i) expression changes in livers of mice treated with arsenic [24], (ii) expression changes identified in arsenic-induced tumors resulting from in utero exposures to arsenic in mice [6], and (iii) expression changes in blood from a human population from Taiwan exposed to arsenic [25]. These comparisons identify overlap of similarly modulated transcripts in response to arsenic exposure that include: *BCL6* (B-cell CLL/lymphoma 6), *CD14* (CD14 antigen), *CXCL1* (chemokine ligand 1), *EGR1* (early growth response 1), *FOS* (v-fos FBJ murine osteosarcoma), *FOSB* (FBJ murine osteosarcoma viral oncogene homolog B), *GADD45B* (growth arrest and DNA damage inducible beta), *IFNGR1* (interferon gamma receptor 1), *IL1B* (interleukin 1 beta)*, IL1R1* (interleukin 1 receptor 1)*, JUN* (v-jun sarcoma virus oncogene), *MAPK6* (mitogen-activated protein kinase 6), *MT1X* (metallothionein 1X), *RAD23B* (RAD23 homolog B), and *TOP1* (topoisomerase DNA 1) (Tables S3 and S5). These results highlight the modulation of stress related transcripts in both mice (acute and in utero exposures) and a separate adult human population in response to arsenic exposure.

## Discussion

Globally, millions of people are at risk for the detrimental effects of chronic arsenic exposure with drinking water levels far exceeding the WHO guideline [1]. Prenatal arsenic exposure in human populations has been associated with pronounced long-term health consequences [4]. Here, we address the impact of maternal arsenic exposure on fetal gene expression in a human population. Our goals were 2-fold: first, to establish the extent to which chronic arsenic exposure in mothers impacts newborn gene expression, and second, to identify genes that could be used as potential biomarkers of prenatal arsenic exposure and targets for remedial therapy.

Differential expression testing of training populations of newborns whose mothers had varied exposures to arsenic identified three arsenic-associated gene expression signatures comprised of 170, 38, and 11 genes. Analysis of the predictive capacity of each of these gene sets using the Support Vector Machine two-class prediction algorithm showed that each of these gene sets is highly predictive of arsenic exposure in a test population. Notably, even the smallest gene set comprised of 11 genes was powerful, with 83% accuracy in predicting prenatal arsenic exposure in the test population. The 11 potential biomarkers of prenatal arsenic exposure include *CXL1*, *DUSP1*, *EGR-1*, *IER2*, *JUNB*, *MIRN21*, *OSM*, *PTGS2*, *RNF149*, *SFRS5*, and *SOC3.* The set of 11 genes show a striking dose response to prenatal arsenic exposure. Stress response and cell cycle regulation are associated molecular functions of the potential biomarker set. Arsenic exposure is known to activate stress-related transcripts in yeast, animal models and human subjects [24–26]. Here, we find that stress-response genes are differentially expressed among a population of newborns whose mothers were exposed to varying levels of arsenic.

To assess the genome-wide impact of prenatal arsenic exposure on newborn gene expression, we identified all transcripts that showed differential expression between two populations; the 21 newborns whose mothers had been exposed to arsenic versus the 11 newborns whose mothers were unexposed. These analyses identified ∼450 genes differentially expressed between the two populations, of which 90% had expression levels that were increased (rather than decreased) by arsenic exposure. Clearly, there is a robust genome-wide response to prenatal arsenic exposure with ∼3% of the expressed genes significantly altered in the newborn. Gene ontology and GSEA highlight the activation of stress-related transcripts in the cord blood of infants exposed prenatally to arsenic.

Furthermore, integration of the gene products of the ∼450 transcripts with known molecular interactions identified the existence of a large arsenic-modulated interacting network of 105 proteins. Embedded within this large interacting network are three sub-networks that highlight that prenatal arsenic exposure activates inflammation-related molecules. Specifically, the first of the sub-networks centers around NF-κB and IL1-β. NF-κB regulates a large number of genes critical for apoptosis, as well as inflammation-related molecules such as cytokines (interleukins). IL1-β belongs to the class of acute phase proteins known to be increased in response to inflammation. Links between prenatal arsenic exposure and the activation of a stress response are also evident in the second and third sub-networks. Prenatal arsenic exposure resulted in the induction of the stress-related transcription factors STAT1 and HIF-1α, both of which are known to be activated by arsenic in model systems [19]. Here, we identify STAT1 and HIF-1α activation in newborn cord blood upon prenatal arsenic exposure. The activation of stress-response proteins such as interleukin-8 (IL-8) in response to prenatal arsenic exposure is also evident in sub-network three. The gene expression signatures identified here as modulated by prenatal arsenic exposure were compared to arsenic-induced gene expression changes in the mouse model and also with a separate human population. These comparisons highlight the common pattern of activation of stress-related transcripts in response to arsenic exposure.

Additionally, eight of the 11 biomarker gene products were found to have significant interactions with the proinflammatory cytokine TNF-α. Several studies in animal models have shown that arsenic exposure results in TNF-α stimulation [27–29]. In this study, TNF-α activation is identified in newborn cord blood upon prenatal arsenic exposure. Taken together, the network findings underscore that a mother's arsenic exposure results in a robust response in the fetus, indicative of a systemic inflammatory response along with the modulation of numerous other biological processes including apoptosis, signal transduction, cell adhesion, and transcription.

We further show that the extensive genome-wide newborn response to prenatal arsenic exposure may be regulated by at least three transcription factors. Analysis of the promoter regions of the arsenic-modulated genes showed enrichment for NF-κB and SRF in all four arsenic-modulated gene sets. SRF transcriptionally activates the expression of immediate early response genes, including *C-FOS* and *EGR-1* [30], two members of the potential arsenic biomarker set. Moreover, binding sites for the metal-responsive transcription factor-1 (MTF1) are enriched in three of the four gene sets (sets 1, 3, and 4). MTF1 was shown to be activated upon arsenic exposure in animal models [23,24]. That gene targets for a known arsenic-inducible transcription factor are found among the transcripts modulated in the cord blood of infants born to arsenic exposed mothers supports our conclusions that the transcriptional changes reported here are likely due to prenatal arsenic exposure.

Our findings clearly demonstrate the robust impact of a mother's arsenic consumption on gene expression in utero as evidenced by transcript levels in the newborn's cord blood. More specifically, our data suggest that prenatal arsenic exposure acts as an inflammatory stimulus that activates the NF-κB signaling cascade. NF-κB activation plays a critical role in inflammation-driven tumor progression [31], and thus key players in tumor progression are modulated in the blood of newborns exposed to arsenic. To determine the extent to which these exposures and the resultant expression changes are associated with susceptibility to disease in later life, the health status of these children is currently being followed.

### Conclusions

In summary, class prediction algorithms identified gene expression signatures that predict arsenic exposure in a test population with about 80% accuracy. Notably, by integrating training populations with varied exposures, a highly predictive potential biomarker gene set composed of just 11 genes was identified. These genes are promising as genetic biomarkers for prenatal arsenic exposure. Currently, we cannot eliminate the possibility that the gene expression signatures identified here are not absolutely specific for arsenic; they may also be predictive of other environmental exposures, e.g., exposure to other heavy metals. Nevertheless, this study underscores that there is a robust prenatal response that correlates with arsenic-exposure levels that could modulate numerous biological pathways including apoptosis, cell signaling, the inflammatory response, and other stress responses, and ultimately affect health status. Arsenic contamination of the drinking water in the Ron Pibul area of Thailand is representative of that seen in many other areas of South East Asia, most notably Bangladesh [9], suggesting that prenatal exposures are likely to be endemic in these areas. Moreover, arsenic contamination of the Ron Pibul drinking water is roughly the same as that known to be present in many of the western United States [2,9], suggesting that prenatal arsenic exposure may also be a problem in the United States. These data contribute to our understanding of biological responses upon arsenic exposure, and show that prenatal exposure in humans results in measurable phenotypic responses in the newborn.

## Materials and Methods

### Study locations and subjects.

The study was conducted in Bangkok and the Ron Pibul District of the Nakhon Sri Thammarat Province located in the southern peninsula of Thailand (Figure S1). Five villages in the Ron Pibul district were selected for the study location as they had been classified as high level arsenic contaminated areas, and arsenicosis had been reported there [8]. Arsenicosis has not been reported in Central Thailand, specifically Bangkok, where arsenic concentrations in water and soil have been determined to be very low [8]. The study subjects consisted of 32 pregnant women (20–40 y old). All subjects were healthy, pregnant volunteers undergoing vaginal childbirth without birth stimulation or anesthesia. Twenty-three pregnant women living in the Ron Pibul District and nine women living in Bangkok for at least 1 y were recruited for the study. Women from both sites were age, educational level, and socioeconomically matched. Questionnaires were administered to all participants to obtain personal information regarding residential history, health history and potential confounding factors, birth and pregnancy information (number of births, abortions or complications), use of community drinking water and well water, plus water and food consumption habits. Cord blood samples were collected from January 2004 to December 2005 in the Ron Pibul Hospital (Ron Pibul District) and the Rajvithi Hospital (Bangkok). This study was conducted according to the recommendations of the Declaration of Helsinki (World Medical Association 1989) for international health research. All subjects gave written informed consent to participate in this study.

### Sample collection and arsenic measurement.

Pregnant participants were asked to provide toenail samples during pregnancy for analysis of total arsenic concentration, which was determined by Inductively Coupled Plasma-Mass Spectrometry (ICP-MS) (Agilent 7500c). After delivery, 2.5 ml of newborn cord blood was collected into a PAXgene Blood RNA (Qiagen) tube for study of gene expression. All cord blood samples were kept at −70 °C until analysis.

### Microarray analysis.

Total RNA was isolated from 32 cord blood samples according to the PAX gene protocol and Qiagen RNA extraction kit. RNA was labeled using a globin reduction protocol (Affymetrix) and hybridized to HGU133 Plus 2.0 full genome human arrays in technical duplicate for a total of 64 arrays. Data were first normalized using Robust Multi-Chip Average (RMA) [32] and filtered for expressed transcripts across all arrays (+2 standard deviations above mean background) resulting in reduction of the probesets from the original 54,675 to 15,265. A mean absolute expression value was calculated from technical duplicates of the arrays for all expressed transcripts. Differential gene expression and association with increasing arsenic concentration was calculated as follows. The samples comprising the training sets were separated into two groups based on arsenic exposure level. The two groups were unexposed (maternal toenail <0.5 μg/g) or exposed (maternal toenail ≥0.5 μg/g). The two-class exposure designation is based on the WHO standards for exposure to arsenic of 10 μg/l arsenic. A mean toenail arsenic concentration of 0.5 μg/g corresponding to chronic consumption of drinking water at 10 μg/l arsenic was derived from two studies associating arsenic toenail concentration and drinking water in a population from Bangladesh [12] and the United States [11]. Differential expression was determined as a significant difference in the expression of a gene (exposed versus unexposed) where the average fold change was greater than +/−1.5 and *p* < 0.05 (*t*-test). Additionally, significant association of gene expression and increasing arsenic level was determined by correlation measurements (*r* ^2^ ≥ +0.6, *r* ^2^ ≤ −0.6; *p* < 0.01) calculated using the linear regression model in S-PLUS 7.0 (http://www.insightful.com). The two-class prediction model used for assessing arsenic exposure in test populations was Support Vector Machine, carried out in Gene Pattern Software (version 2.0.1) (http://www.broad.mit.edu). Multivariate analysis was performed as follows: the expression values (*Y*) for each gene were modeled using *Y =* β_1_ + β_2_ *ars* (arsenic) *+* β_3_ *loc* (geographic location), where toenail arsenic concentration is a continuous variable and location is binary. Statistical significance was determined by subjecting β_2_ and β_3_ to *t*-statistics. A χ^2^ test for dependence (association) of the two factors (e.g., arsenic and geographic location) was performed for the set of arsenic biomarkers. A Fisher's exact test was employed to determine overrepresentation of the biomarkers within the genes significantly associated with either geographic source or arsenic exposure (*p* < 0.01). Network analyses were performed using the Ingenuity software (http://www.ingenuity.com). Gene ontology enrichment analysis was performed using GO Miner [33]. GSEA [34] was performed using the GSEA desktop software [35], with a false discovery rate correction (Benjamini-Hochberg) employed. Microarray data have been deposited to the Gene Expression Omnibus repository.

### Transcription factor binding site analysis.

Transcription factor binding site analysis was performed using Expander software [36] and Genomatix software (http://www.genomatix.de). For both analyses, Affymetrix probesets were linked to sequence data for regions 1,000 base pairs upstream and 200 base pairs downstream of the transcription start sites, and these were analyzed for significant enrichment of transcription factor binding sites. Significance (*p* ≤ 0.05) was calculated where significance is the probability of obtaining an equal or greater number of sequences with a model match in a randomly drawn sample of the same size as the input sequence set.

## Supporting Information

Figure S1 Map of Study Location The study was conducted in Bangkok and the Ron Pibul District of the Nakhon Sri Thammarat Province located in the southern peninsula of Thailand. Study locations are indicated with red circles.
(6.3 MB AI).

Figure S2 Expression Patterns of 11 Biomarkers of Prenatal Arsenic Exposure Transcripts that are predictive of prenatal arsenic exposure are plotted with expression intensity versus arsenic exposure.
(615 KB AI).

Figure S3 Arsenic-Modulated Sub-networks Identified from Differentially Expressed Genes from the Ron Pibul Population Significant sub-networks of arsenic-modulated gene products were identified between the unexposed and exposed newborn populations of Ron Pibul.
(10.9 MB AI).

Table S1 Genes Differentially Expressed between Newborns That Composed the First Training Population A total of 170 genes were differentially expressed between newborns born to mothers unexposed to arsenic and newborns born to mothers exposed to arsenic that composed the first training population.
(54 KB DOC)

Table S2 Genes Differentially Expressed between Newborns That Composed the Second Training Population A total of 38 genes were identified as differentially expressed between newborns born to mothers unexposed to arsenic and newborns born to mothers exposed to arsenic that composed the second training population.
(29 KB DOC)

Table S3 Genes Differentially Expressed between Two Newborn Populations; Those Born to Mothers Unexposed to Arsenic and Those Born to Mothers Exposed to Arsenic A total of 447 genes were identified as differentially expressed between the two newborn populations; those newborns born to mothers unexposed to arsenic or to mothers exposed to arsenic.
(122 KB DOC)

Table S4 Arsenic-Modulated Gene Products A total of 105 arsenic modulated gene products are contained in a significant large interactome (*p* < 10^−55^).
(38 KB DOC)

Table S5 Genes Differentially Expressed between Arsenic-Unexposed and -Exposed Newborns in Ron Pibul A list of the 321 genes differentially expressed between the two newborn populations from the Ron Pibul Province; those born to mothers unexposed to arsenic, and those born to mothers exposed to arsenic.
(85 KB DOC)

### Accession Numbers

Microarray data have been deposited to the National Center for Biotechnology Information (NCBI) Gene Expression Omnibus repository under Series Record GSE7967 (http://www.ncbi.nlm.nih.gov/geo/).

## Abbreviations

FDR: false discovery rate
GSEA: Gene Set Enrichment Analysis
WHO: World Health Organization
