# Supplementary material for: Activation of Inflammation/NF-κB Signaling in Infants Born to Arsenic-Exposed Mothers
Source: PLoS Genet. 2007 Nov 23;3(11):e207. doi: 10.1371/journal.pgen.0030207 (PMC2082467; doi:10.1371/journal.pgen.0030207)
Supplement: Supplementary file 1 [file pgen.0030207.sg001.pdf]

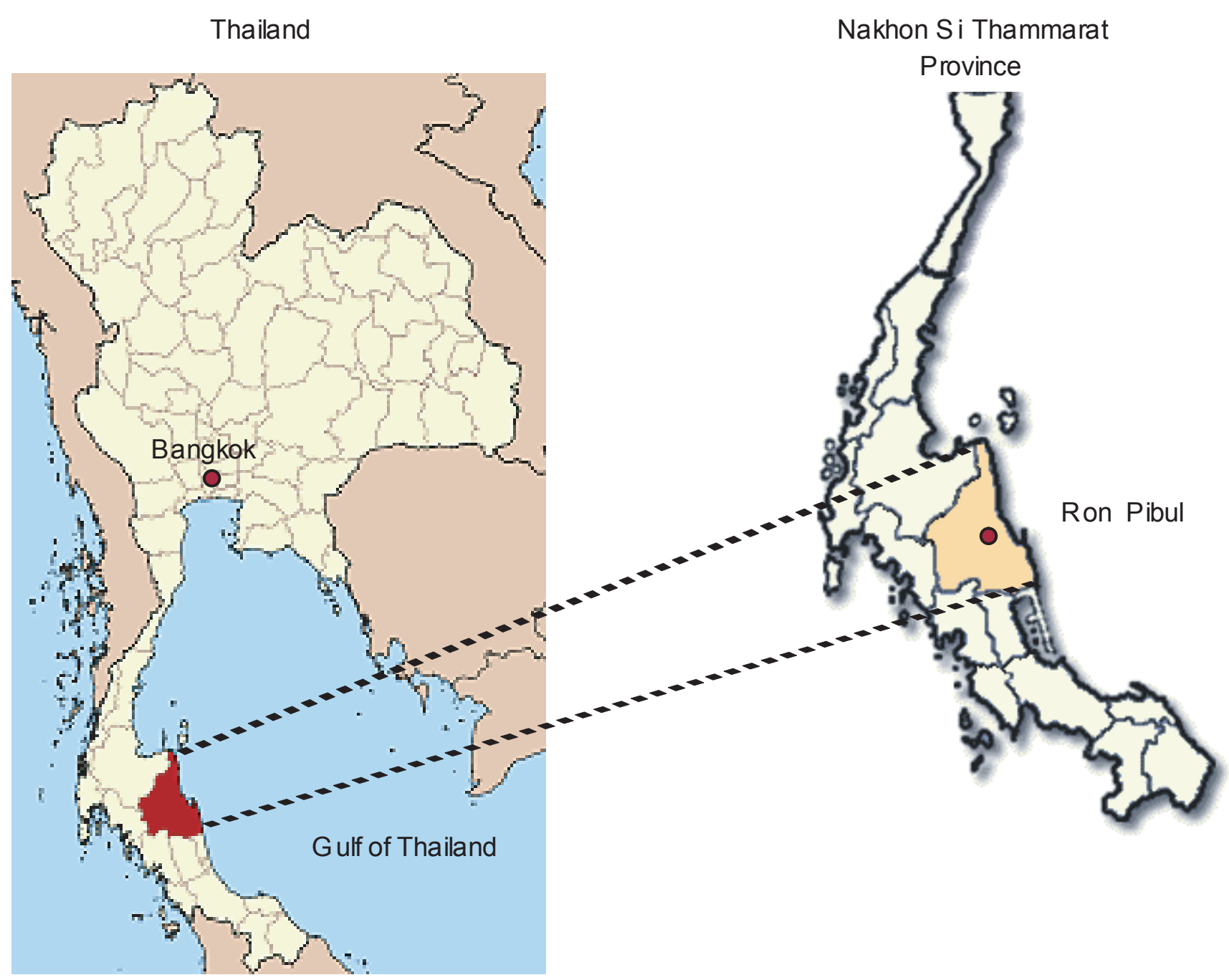

Figure S1. Map of study site. The study was conducted in Bangkok and the Ron Pibul District of the Nakhon Sri Thammarat Province located in the southern peninsula of Thailand. Study locations are indicated in red.
