# Supplementary material for: Activation of Inflammation/NF-κB Signaling in Infants Born to Arsenic-Exposed Mothers
Source: PLoS Genet. 2007 Nov 23;3(11):e207. doi: 10.1371/journal.pgen.0030207 (PMC2082467; doi:10.1371/journal.pgen.0030207)
Supplement: Supplementary file 2 [file pgen.0030207.sg002.pdf]

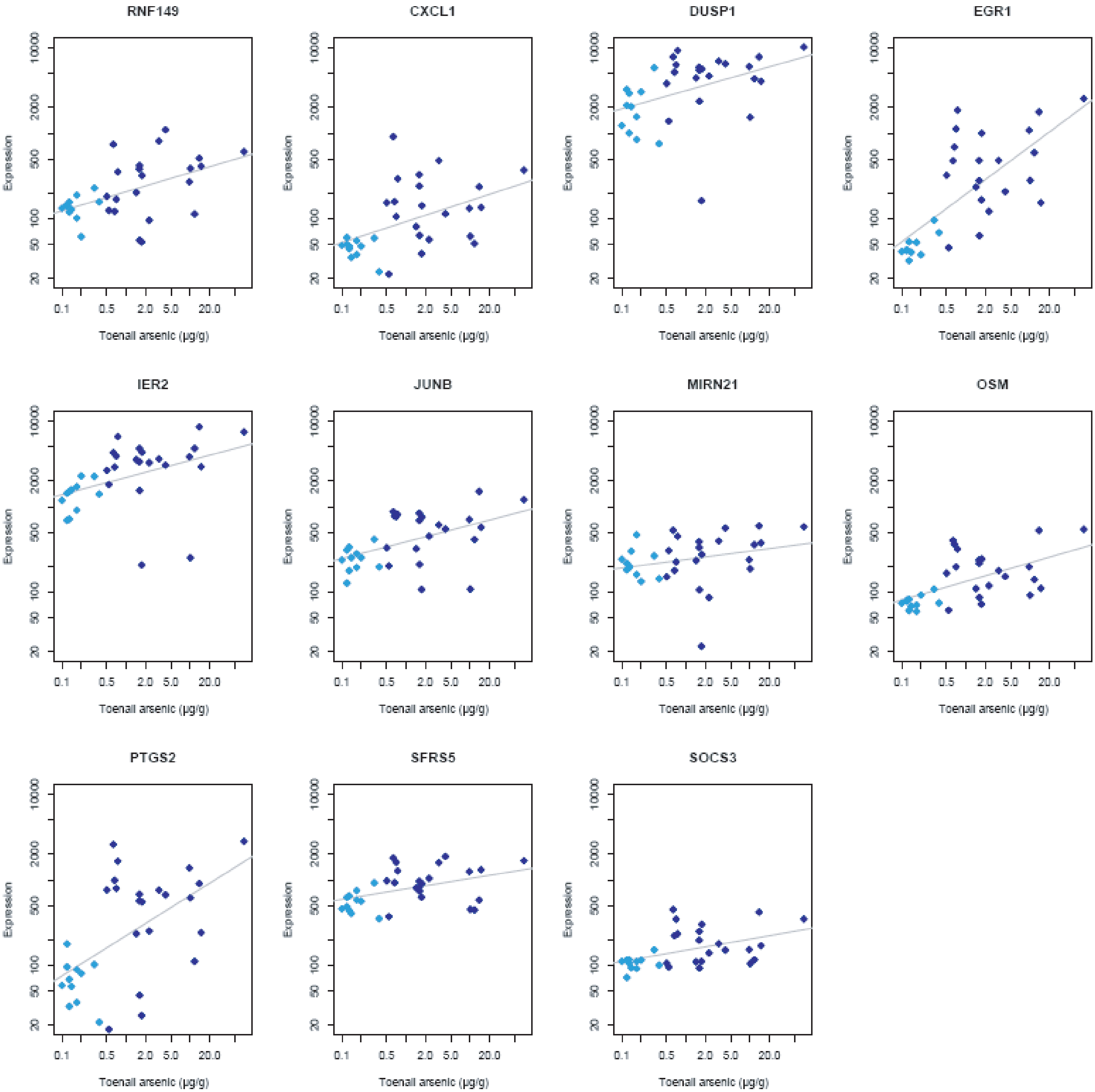

Figure S2. Expression patterns of the 11 potential biomarkers of prenatal arsenic exposure. Absolute gene expresion levels are plotted versus toenail arsenic concentration. Circles represent each of the 32 subjects of the population with light blue representing the arsenic unexposed subjects and dark blue representing the arsenic exposed subjects.
