# Supplementary material for: Activation of Inflammation/NF-κB Signaling in Infants Born to Arsenic-Exposed Mothers
Source: PLoS Genet. 2007 Nov 23;3(11):e207. doi: 10.1371/journal.pgen.0030207 (PMC2082467; doi:10.1371/journal.pgen.0030207)
Supplement: Supplementary file 3 [file pgen.0030207.sg003.pdf]

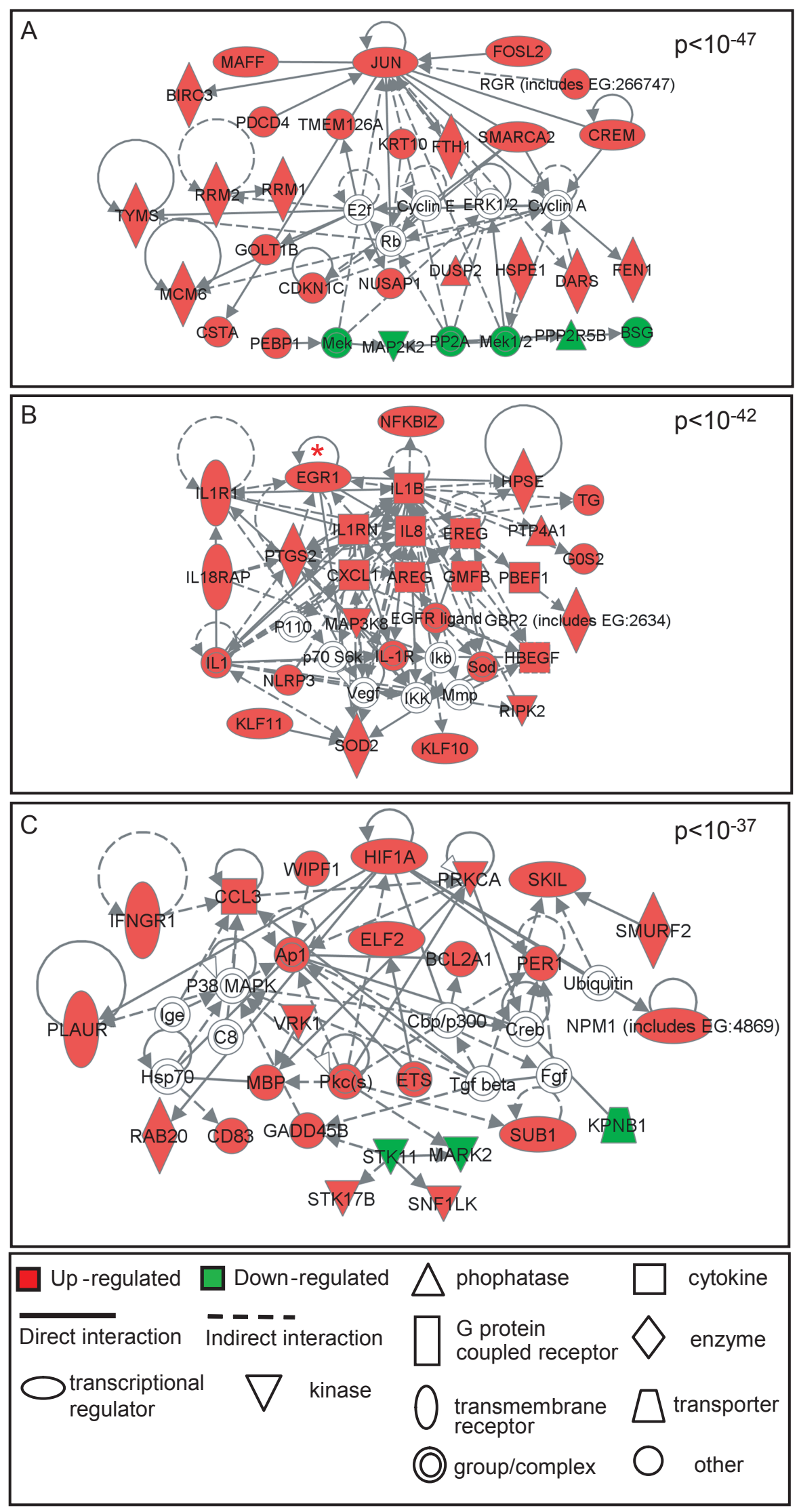

Figure S3. Sub-networks of prenatal arsenic-modulated gene products identified from the Ron Pibul population.

(A) A sub-network that integrates JUN was identified. (B) A sub-network that integrates NF-κB1 with EGR-1 was identified. Note that EGR-1 is among the 11 potential gene biomarkers for arsenic exposure shown in Table 1. (C) A Hif1-α focused sub-network was identified. Proteins encoded by 11 potential gene biomarkers for arsenic exposure are indicated with (*). Proteins in red represent arsenic-induced transcripts, proteins in green represent arsenic-repressed transcripts.
